# Signaling Pathways and Key Genes Involved in Regulation of foam Cell Formation in Atherosclerosis

**DOI:** 10.3390/cells9030584

**Published:** 2020-03-01

**Authors:** Anastasia V. Poznyak, Wei-Kai Wu, Alexandra A. Melnichenko, Reinhard Wetzker, Vasily Sukhorukov, Alexander M. Markin, Victoria A. Khotina, Alexander N. Orekhov

**Affiliations:** 1 Institute for Atherosclerosis Research, Skolkovo Innovative Center, Moscow 121609, Russia; sasha.melnichenko@gmail.com; 2 Department of Internal Medicine, National Taiwan University Hospital, Bei-Hu Branch, Taipei 10617, Taiwan; weikaiwu0115@gmail.com; 3 Laboratory of Angiopathology, Institute of General Pathology and Pathophysiology, Moscow 125315, Russia; nafany905@gmail.com; 4 Clinic for Anesthesiology and Intensive Care Medicine, University Hospital Jena, Am Klinikum 1, D-07747 Jena, Germany; Reinhard.Wetzker@uni-jena.de; 5 Institute of Human Morphology, Moscow 117418, Russia; vnsukhorukov@gmail.com (V.S.); alexander.markin.34@gmail.com (A.M.M.)

**Keywords:** atherosclerosis, foam cells, CVD, LDL, modified LDL

## Abstract

Atherosclerosis is associated with acute cardiovascular conditions, such as ischemic heart disease, myocardial infarction, and stroke, and is the leading cause of morbidity and mortality worldwide. Our understanding of atherosclerosis and the processes triggering its initiation is constantly improving, and, during the last few decades, many pathological processes related to this disease have been investigated in detail. For example, atherosclerosis has been considered to be a chronic inflammation triggered by the injury of the arterial wall. However, recent works showed that atherogenesis is a more complex process involving not only the immune system, but also resident cells of the vessel wall, genetic factors, altered hemodynamics, and changes in lipid metabolism. In this review, we focus on foam cells that are crucial for atherosclerosis lesion formation. It has been demonstrated that the formation of foam cells is induced by modified low-density lipoprotein (LDL). The beneficial effects of the majority of therapeutic strategies with generalized action, such as the use of anti-inflammatory drugs or antioxidants, were not confirmed by clinical studies. However, the experimental therapies targeting certain stages of atherosclerosis, among which are lipid accumulation, were shown to be more effective. This emphasizes the relevance of future detailed investigation of atherogenesis and the importance of new therapies development.

## 1. Introduction

Atherogenesis is a complex process that involves numerous cellular mechanisms and pathways, such as inflammation, lipid accumulation, oxidative stress, and others. Our understanding of the processes underlying atherosclerosis is constantly improving. Over the past decades, clinical and basic research provided evidence of the immune system involvement at all stages of atherosclerosis, from lesion initiation and progression to the advanced plaque formation and destabilization precipitating acute clinical symptoms.

Atherosclerosis is the underlying condition of most of the cardiovascular diseases (CVD) that remain the leading cause of adult morbidity and mortality worldwide [1,2]. American Heart Association reported that, in 2017, 17.9 million deaths were caused by CVD, which is 14.5% more than recorded in 2006. CVD was found to be accountable for 31% of all deaths in the world. These data position atherosclerosis as not only clinical, but also a major socioeconomic problem [3]. Correspondingly, atherosclerosis remains an attractive research topic, and the search for novel anti-atherosclerosis therapies continues.

According to current understanding, both resident blood vessel wall and circulating immune cells are involved in atherosclerosis progression. Furthermore, such factors as genetics, hemodynamics of the blood flow and plasma lipoproteins have an impact on the disease progression, and the interplay of aforementioned aspects may form atherosclerotic clinical pattern [4,5].

In this review, we focus on foam cells that are crucial for atherosclerosis lesion formation. It has been demonstrated that the formation of foam cells (cellular lipidosis or cellular lipid retention) is induced by modified low-density lipoprotein (LDL) [6].

The concept of the inflammatory nature of atherosclerosis pathogenesis promoted testing of a range of drugs for anti-atherosclerotic activity. Anti-inflammatory and antioxidant drugs were investigated for their potential to reverse or at least slow down the atherogenic processes. Many of them, however, failed clinical trials. For example, methotrexate could not reduce cardiovascular events [7], beta carotene did not lower the cardiovascular risk [8], and vitamin E did not lower the number of cardiovascular-associated deaths even in combination with vitamin C and/or β-carotene [9,10].

By contrast, some promising results were obtained in the CANTOS (Canakinumab Anti-Inflammatory Thrombosis Outcomes Study) clinical trial that demonstrated the efficacy of canakinumab (150 mg), a monoclonal antibody that inhibits inflammation by neutralizing Interleukin-1β, for reducing the primary endpoint: non-fatal myocardial infarction (MI), stroke, or cardiovascular death [11]. Thanks to these results, inflammation regained the attention as possible point of therapeutic intervention for atherosclerosis treatment. However, it appeared to be more promising to target specific proinflammatory pathways rather than inflammation in general [12,13,14]. 

The experimental therapies targeting certain stages of atherosclerosis, among which are lipid accumulation, were shown to be more effective than unspecific anti-inflammatory treatments. This emphasized the relevance of future detailed investigations of atherogenesis and the importance of new therapies development [15].

## 2. The Origin of Foam Cells

Foam cells are characterized by multiple lipid inclusions in the cytoplasm that gives them a specific look reflected by the commonly used name. The emergence of the foam cells in the arterial intima is considered to be one of the earliest manifestations of atherosclerosis, referring to the preclinical atherogenesis. Moreover, the underlying extra- and intracellular lipid deposition is a crucial trigger of atherosclerotic lesion development [16,17].

It has long been considered that all foam cells of human atherosclerotic lesions derive exclusively from macrophages and monocytes. This misconception was based on the results coming from studies on murine models, in which macrophages represent a prevalent source of foam cells. However, studies of human arteries demonstrated the prominent role of resident cell types present in the arterial wall, among which are vascular smooth muscle cells (VSMCs) and pericytes, or stem/progenitor cells (SPCs) [18,19]. The numbers of macrophages and monocytes present in the subendothelial intima of unaffected human arteries are not high, accounting for only 3–5% of total cell population. Since the arterial intima is the main site of foam cell formation, vascular smooth muscle a-actin (SMA)-positive resident cells (typical smooth muscle cells and pericyte-like cells) came into focus [20,21]. Recent studies clearly linked the SMA-positive cells with both foam cells formation and lipid accumulation, indicating that pericytes may be the leading source of foam cells [22,23]. Macrophages are now considered to be the second biggest source, and the minor role of other cell types is also known. However, macrophage cultures still appear to be the most representative model of foam cell formation and continue to be widely used. 

## 3. Foam Cells in Atherosclerosis

Internalization of lipid particles by macrophages in the subendothelial space is mediated by a range of scavenger receptors (SRs), each of which complies with an exact LDL modification. For example, receptors of the SR-A class, SR-AI and SR-AII, have a strong affinity for acetylated LDL and extensively oxidized LDL [24]. Noteworthy, SRs are not the only way for macrophages to uptake modified LDL, and phagocytosis and pinocytosis can also take place [25]. 

Atherogenesis is associated with serious disruption of lipid metabolism, including both increased cellular cholesterol uptake and reduced cholesterol efflux. Potential causes of these alterations may be represented by upregulation of the SRs that mediate modified LDL binding. By contrast, inhibition of the expression of cholesterol pumps results in normalization of cholesterol efflux [26,27]. This hypothesis is at least in part supported by the finding that modified LDL-induced cholesterol accumulation in human macrophages of monocyte origin was accompanied by the upregulation of proinflammatory genes [28]. Nevertheless, it remains to be established whether the proinflammatory response can be considered as a trigger of foam cells formation [29]. A couple of recent papers report that the proinflammatory response is promoted by intracellular lipid accumulation [30,31].

**Figure 1:**
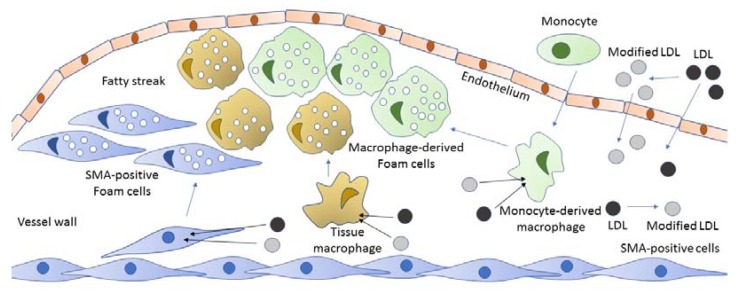
From the blood flow, both LDL and modified LDL enter the vessel wall, where they can be internalized by macrophages, pericytes, and vascular smooth muscle cells (vascular smooth muscle a-actin (SMA-positive cells)) via scavenger receptors or by phagocytosis or pinocytosis. These macrophages and SMA-positive cells with the taken-up lipid contents in their cytoplasm become foam cells that, in turn, accumulate in the vessel wall and form fatty dots and streaks—an initial stage of the atherosclerotic lesion.

Newly formed foam cells accumulate in the arterial intima and initiate the formation of atherosclerotic lesions, which at the early stages are called fatty streaks [32]. This process is briefly summarized in Figure 1.

## 4. Role of Modified LDL in Foam Cells Formation

Even though both macrophages and SMA-positive cells are able to internalize native LDL, the majority of lipids taken up during foam cells formation are coming from modified LDL. Studies in cultured cells indicated the inability of native LDL to cause intracellular accumulation of lipids and the consequent formation of foam cells, while significant accumulation of intracellular lipids was observed when native LDL was replaced with in vitro chemically modified lipoprotein particles [33,34].

Oxidized LDL (oxLDL) has long been considered to be the only type of modified LDL that is significant for atherogenesis, and indirect signs of oxLDL presence in the blood of atherosclerotic patients supported this suggestion [35]. However, all attempts to isolate circulating oxLDL failed [36].

A hypothesis of multiple modifications of the same LDL particle was formulated based on the properties of several LDL types that could be isolated and characterized by physical-chemical methods. At least three modified atherogenic LDL forms have been observed, namely small dense LDL, electronegative or LDL(–), and desialylated LDL, each of which was shown to possess specific features, including susceptibility to oxidation [37,38].

Noteworthy, LDL oxidation occurs at the very end of the modification cascade, which is confirmed by the experiment in which LDL isolated from healthy individuals was incubated with plasma-derived serum obtained from atherosclerotic patients. After 1 h of incubation of this mixture at 37 °C, LDL became atherogenic, and presence of desialylated LDL was observed. After 6 h, lipid content and particle size appeared to decrease and particles became more electronegative. Only after incubation for 48–72 h, a decrease of the antioxidant content and increase of LDL susceptibility to oxidation was observed [39]. 

Several mechanisms explaining the atherogenic properties of modified LDL have been described, including: (1) self-association of LDL particles; (2) LDL association with the extracellular matrix components; and (3) formation of LDL-containing immune complexes. 

It was shown that desialylated LDL is characterized by the enhanced cellular uptake of particles characterized by a slower degradation rate within the cells. That may explain the increased atherogenicity of this type of modified LDL. Association of modified LDL particles with collagenase-resistant arterial debris, collagen, elastin, and proteoglycans was shown in human aortic intima. This also promoted cholesterol accumulation in cultured cells. On the contrary, isolated electronegative LDL showed no ability to promote foam cells formation by itself, but its atherogenicity was enhanced by the high susceptibility to association [40]. 

Another interesting type of complexes isolated from the blood of atherosclerotic patients is represented by LDL-containing circulating immune complexes that promote cholesterol accumulation in cultured cells more potently than multiple modified LDL alone. Moreover, the atherogenic effect of these complexes was also increased by their ability to induce proinflammatory cytokines secretion and promote apoptosis in macrophages [41,42].

## 5. Key Genes and Signaling Pathways Involved in Foam Cells Formation

One of the most intriguing aspects of foam cells formation is the cellular signaling and interaction of genes that is especially important because of its potential significance for identification of novel diagnostic markers and therapeutic targets.

Currently, signaling pathways involved in lipid accumulation are well known and have been thoroughly reviewed [43,44,45,46]. CD36 is a highly specific receptor to oxLDL, which participates in foam cell formation. Activated by oxLDL, CD36 induces a signaling cascade that up-regulates the expression of CD36 and thus facilitates the uptake of oxLDL. The interaction between CD36 and oxLDL promotes phosphorylation of Src kinases that, in turn, activate the mitogen-activated kinases Jun-kinase (JNK) 1 and 2 and VAV, a guanine nucleotide exchange factor, which mediates the uptake of oxLDL [43]. Moreover, the CD36-dependent lipid accumulation in macrophages is up-regulated through Wnt5a activation via Fz5-dependent signaling [44] and by PPARγ activation via p38 MAPK signaling [45].

It is well established that Toll-like receptors (TLRs) signaling plays an important role in atherosclerosis pathogenesis and foam cell formation [46]. TLR2 activates by CD36, co-expresses with Wnt5a, and, apparently, participates in foam cell formation [46]. TLR4 may participate in lipid accumulation via PI3K/mTORC2 pathway, which is referred to as the AKT phosphorylation [47]. Moreover, it has been demonstrated, recently, that TLR4-dependent signaling drives extracellular catabolism of aggregated LDL. Macrophages via TLR4/MyD88 (myeloid differentiation primary response 88)/PI3K (phosphoinositide 3-kinase)/SYK (spleen tyrosine kinase)/AKT-dependent signaling pathway make the lysosomal synapse degrade the aggregated LDL and accumulate the lipids. Thus, TLR4 signaling intermediates foam cell formation [48]. TLR9 may provide foam cell formation in an IRF7- and NF-κB-dependent manner [49] and via the p38 MAPK pathway [50].

A recent study has reported 10 master-regulator genes that could be the main regulators of the foam cells formation: IL7R, TIGIT, ITIM, CXCL8, F2RL1, EIF2AK3, IL7, TSPYL2, ANXA1, DUSP1, and IL15 [40]. Notably, nine of these ten genes were associated with the molecular and cellular mechanisms of atherosclerosis and comorbid conditions (e.g., hypertension, diabetes, and metabolic syndrome). Seven of the ten genes were involved in the inflammatory pathway, consistent with previous findings [51,52]. However, the most unexpected and crucial finding was the fact that none of the identified genes participated in the cholesterol metabolism pathways. Previously, it was assumed that changes in the regulation of genes result from the accumulation of lipids in the arterial cells [30]. It is likely that the opposite is true: non-intracellular accumulation of cholesterol causes a reaction of innate immunity, but the immune response due to the interaction of cells with modified LDL stimulates the accumulation of intracellular lipids. It is very important to find out what occurs first: accumulation of intracellular lipids or the proinflammatory reaction.

Several recent studies investigated the role of mollified LDL [53,54]. Native LDL, multiple modified LDL isolated from the blood of atherosclerotic patients, and LDL modified in vitro by desialylation, oxidation, and acetylation were used. In addition, latex beads as a stimulator of macrophage phagocytotic activity were added to cultured human monocyte-derived macrophages. It was shown previously that any type of LDL modification causes a tendency to self-association of lipoprotein particles [55,56]. The sizes of the associates do not allow them to interact with a specific LDL receptor. As a result, associates are taken up by nonspecific phagocytosis bypassing the LDL receptor. It is important to establish whether there are common mechanisms in the interaction of macrophages with associates of modified LDL and non-lipid latex particles of a similar size.

**Figure 2:**
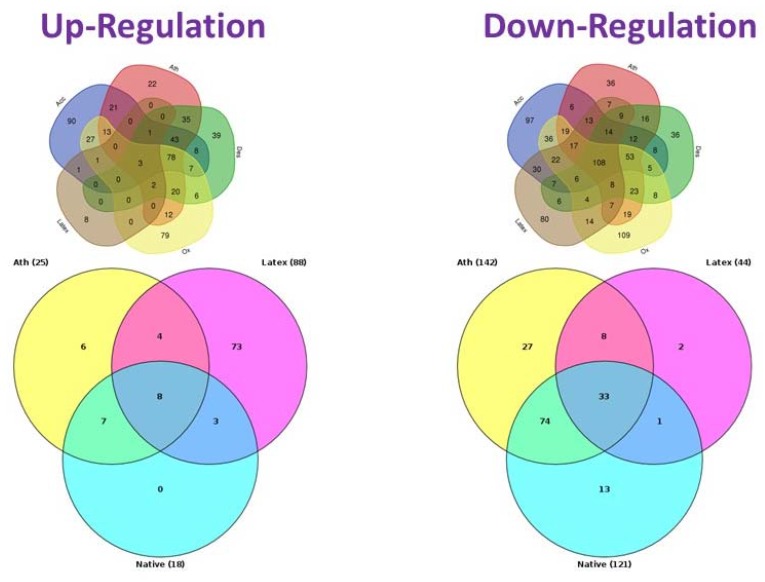
Venn diagrams of signaling pathways. The upper panel shows all the tested samples (Ath, atherogenic LDL; Acc, acetylated LDL; Des, desialyated LDL; Ox, oxidized LDL; Latex, latex beads). The bottom panel show only signaling pathways that change when interacting with native LDL (control), as well as latex beads and multiple modified LDL isolated from the blood of atherosclerotic patients (reported: [54]).

Figure 2 demonstrates Venn diagrams of signaling pathways that are associated with cholesterol accumulation caused by naturally occurring multiply-modified LDL and latex beads. The study of the mechanisms of intracellular lipid accumulation did not include native LDL that does not cause lipid accumulation. However, some signaling pathways were shown to be regulated by both latex beads and modified LDL in a similar manner. Among these signaling pathways, four underwent up-regulation and eight were down-regulated.

The obtained results indicated that common pathways may exist between latex beads and modified LDL effects. Such pathway could be stimulation of phagocytosis. As is known, the stimulation of phagocytosis is a trigger for the proinflammatory response of the macrophage, which is accompanied by the secretion of proinflammatory cytokines. Perhaps the identified common signaling pathways for latex beads and associates of modified LDL taken up by phagocytosis can be involved in the proinflammatory cellular response (see Table 1).

## 6. Proinflammatory Response as an Inductor of Intracellular Lipid Accumulation

A recent study revealed the genes encoding inflammatory molecules that were up-regulated upon treatment of macrophages with atherogenic modified LDL and were involved in the development of inflammation [40]. Further knock-down experiments elucidated the relationship between the immune response and cholesterol accumulation [53]. Primary human macrophages were cultured with atherogenic naturally occurring multiply-modified LDL that induced a prominent cholesterol accumulation. The knock-down of the EIF2AK3 and IL15 genes completely prevented cholesterol accumulation in cultured macrophages. The knock-down of the ANXA1 gene even caused a significant decrease of the basal cholesterol level in cultured macrophages. It is therefore possible that, as a result of the up-regulation of these genes, production of proinflammatory molecules can influence the intracellular cholesterol accumulation.

The latter possibility was explicitly confirmed by another study. Some interleukins, including IL-34 and IL-32, were recently shown to be related to foam cells formation. Liu et al. in 2018 demonstrated that IL-34 can act as a promotor of the lipid accumulation in bone marrow-derived macrophages through the up-regulation of CD36 expression [30]. Earlier, Xu et al. showed that IL-32 stimulated lipid accumulation in THP-1 macrophages and inhibited the cholesterol efflux, thus facilitating foam cell formation [31]. In addition, our recent data indicate that inflammatory cytokines not only promoted the accumulation of intracellular cholesterol caused by modified LDL, but were also able to induce cholesterol accumulation in cultured primary macrophages (Table 2). IL-6 and IL-15 significantly promoted intracellular cholesterol accumulation caused by atherogenic modified LDL. In addition, IL-6 induced significant accumulation of cholesterol even without LDL addition.

Primary human monocyte-derived macrophages were cultured in serum-supplemented medium with cytokines and multiply-modified LDL isolated from atherosclerotic patients. After 24 h, cellular lipids were extracted and total cholesterol was measured.

**Figure 3:**
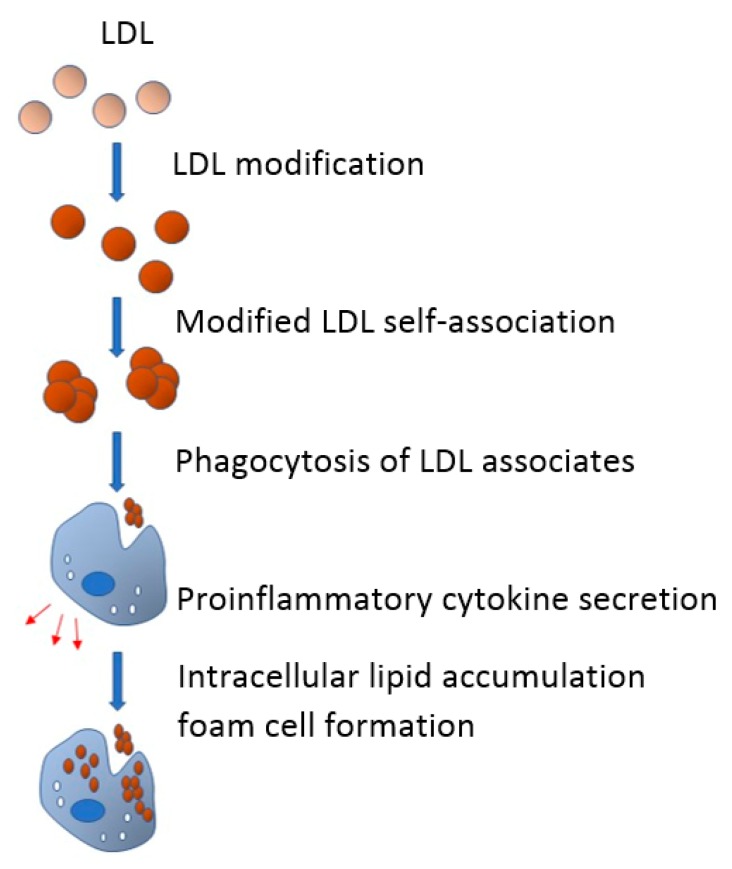
In the blood flow, LDL undergoes multiple modifications and acquires atherogenic properties. Modified LDL particles have a tendency to form self-associates that, in turn, promote phagocytosis of subendothelial arterial cells. Being triggered by phagocytosis, proinflammatory response emerges and proinflammatory cytokines are secreted. Proinflammatory cytokines promote or even cause the accumulation of intracellular lipids, which leads to the formation of foam cells.

According to the revised hypothesis, the proinflammatory response could be an inductor of the intracellular lipid accumulation (Figure 3) [57]. This hypothesis involves not only lipid accumulation and proinflammatory response, but also underlines the crucial role of phagocytosis in the aforementioned relation.

## 7. Conclusions

During recent years, many aspects of atherosclerosis development have been revised, improving the general understanding of the disease. 

First, atherosclerosis was found to be more complex than just a chronic inflammation of the vessel wall in response to damaging stimuli, which was supported by the growing body of evidence about the significance of genetic and metabolic factors.

It was shown that macrophages are not the exclusive source for foam cells formation. Pericytes and VSMCs were also shown to have the ability to transform to foam cells. Moreover, studies of modified LDL helped formulate the hypothesis of the modification cascade that provides a new model of multiple modifications and reduces the significance of oxLDL, which is formed at the end of the modification cascade.

However, perhaps the most crucial update in our understanding of atherogenesis is the conception of the proinflammatory response as an inductor of intracellular lipid accumulation, which appears to be the cause rather than a consequence of atherosclerosis progression. The most recent research on signaling pathways and identification of master-regulator genes helped transform the conception of the consequence of the events that lead to intracellular lipid accumulation. This consequence now has to be presented as follows: associates of modified LDL interact with cells and thus stimulate phagocytosis, which, being the initial event of the innate immunity reaction, induces the release of proinflammatory cytokines that, in turn, cause or enhance intracellular lipid accumulation.

Inflammation and lipid accumulation are considered to be key processes of atherosclerosis development. Thus, the correct causal link between these processes needs to be established. 

All listed updates have the potential to become a basis for effective therapeutic approaches, but further research is needed. The most promising strategy for the development of effective treatment is targeting of particular signaling pathways that underlie one of the crucial stages of the disease. This is the most straightforward approach that leads to identification and design of both effective and safe compounds for atherosclerosis treatment.

## Figures and Tables

**Table 1:** Unidirectional signaling pathways associated with modified LDL and latex beads (reported: [54]).

Direction	Signaling Pathways
up	Neurotrophic signaling TLR2-mediated signaling TLR9 pathway VEGF-A pathway
down	Aurora-B cell cycle regulation Cdc20 deubiquitination Cdc20 ubiquitination CyclinB1 ubiquitination → anaphase onset Fzr1 → cyclin B1 degradation Metaphase to Anaphase transition Securin degradation Usp44 → Cdc20

**Table 2:** Effects of inflammatory cytokines on intracellular cholesterol.

	Intracellular Cholesterol, % of Control	P	
		Vs. Control	vs LDL
**Control**	100.0 ± 21.0	N/A	N/A
+ LDL, 100 µg/mL	162.5 ± 20.3	<0.01	N/A
+ LDL + IL-6, 50 ng/mL	199.5 ± 16.5	<0.01	<0.01
+ IL-6, 50 ng/mL	129.5 ± 8.2	0.01	0.01
+ LDL + IL-15, 50 ng/mL	187.0 ± 13.12	<0.01	<0.01
+ IL-15, 50 ng/mL	107.9 ± 7.3	0,6	<0.01
